# The temporal trends of prevalence and years lived with disability of anaemia in China, Japan, and South Korea, from 1990 to 2021: Results from the Global Burden of Disease Study 2021

**DOI:** 10.7189/jogh.14.04073

**Published:** 2024-05-24

**Authors:** Jie Hu, Zongbin Song, Liang Zhao, Sarel Chavarria Gonzalez, E Wang, Xinran Hou

**Affiliations:** 1 Department of Anesthesiology, Xiangya Hospital, Central South University, Changsha, Hunan, China; 2 Hunan Key Laboratory of Molecular Precision Medicine, Xiangya Hospital, Central South University, Changsha, Hunan, China; 3 National Clinical Research Center for Geriatric Disorders, Xiangya Hospital, Central South University, Changsha, Hunan, China; 4 Department of Hematology, Xiangya Hospital, Central South University, Changsha, Hunan, China

## Abstract

**Background:**

Studies have shown that the disease burden of anaemia varies globally, yet they have not yet determined its exact extent in East Asian countries specifically. We thus aimed to investigate the prevalence and years lived with disability (YLDs) due to anaemia from 1990 to 2021 in China, Japan, and South Korea.

**Methods:**

We extracted the prevalence and YLDs with their age-standardised rates (ASRs) in China, Japan, and South Korea from the Global Burden of Disease Study 2021, stratified by sex, age, and causes. We then examined the temporal trend of anaemia burden from 1990 to 2021 using joinpoint analysis and the association of anaemia burden with the Human Development Index and Universal Health Index through Spearman’s correlation analysis.

**Results:**

In 2021, anaemia affected 136 million people in China (95% uncertainty interval (UI) = 131, 141), with ASRs of prevalence of 8.9% (95% UI = 8.6, 9.3), and accounted for 3.0 million YLDs (95% UI = 2.0, 4.4). It affected 13.6 million people in Japan (95% UI = 11.8, 16.0), with ASRs of prevalence of 7.4% (95% UI = 6.1, 9.0), and caused 181 thousand YLDs (95% UI = 108, 282). It also affected 2.7 million individuals in South Korea (95% UI = 2.4, 3.0), with ASRs of prevalence of 5.2% (95% UI = 4.6, 5.7), and led to 34 thousand YLDs (95% UI = 22, 55). We observed a significant gender discrepancy in the anaemia burden in these three countries, with the prevalence and YLD rates in women being almost twice as high as those in men. Moreover, the peak age of the anaemia burden shifted toward higher age groups in all three countries, particularly in Japan. Chronic kidney disease was responsible for a growing share of anaemia cases and YLDs, especially in adults aged more than 60 years in Japan and South Korea. Haemoglobinopathies were another noticeable cause of anaemia in China, though dietary iron deficiency remained the leading cause. Both socioeconomic development and essential health service coverage showed negative associations with the anaemia burden in the three countries in the past three decades, though with differential patterns.

**Conclusions:**

Anaemia remains a major public health issue in China, Japan, and South Korea; targeted surveillance and interventions are recommended for high-risk populations and cause-specific anaemia.

Anaemia, a disorder characterised by a reduction in haemoglobin or red blood cells [1], is attributed to various aetiologies, including nutritional deficiencies, inflammation, genetic haemoglobin disorders, blood loss, and haemolysis [2]. It has long been a major health concern worldwide due to its associations with unfavourable outcomes, such as increased morbidity and mortality in various populations including neonates [3], children [4], pregnant women [5–7], older adults [8,9], and surgical patients [10,11], as well as decreased work productivity.

The Global Burden of Disease (GBD) study has been the most comprehensive worldwide observational epidemiological study, which provided updated global estimations for hundreds of diseases [12]. Among other diseases, the latest GBD 2021 reported on the global prevalence and years lived with disability (YLDs) caused by anaemia [13]. The epidemiological distribution of anaemia has shown substantial geographical heterogeneity, possibly due to factors such as genetic backgrounds, climate features, and social development. However, few studies have focussed on its epidemiology in East Asian countries, such as China, Japan, and South Korea, which have large populations, unique cultures, and growing economic influence across the world. Despite sharing similar racial, ancestral, and cultural backgrounds, China, Japan, and South Korea differ distinctly in the size and compositions of their populations, as well as their socioeconomic development levels [14]. Considering this, we hypothesised that there should be both similarity and disparity of anaemia burden in the three countries.

With the latest releases of GBD 2021 data on the prevalence and YLD of anaemia prevalence, as well as the Human Development Index (HDI) data from the United Nations (UN) and the Universal Health Coverage (UHC) data from the World Health Organization (WHO), we attempted to fill this gap in knowledge by establishing an up-to-date overview of the disease burden of anaemia, stratified by gender, age, and causes; and by analysing its temporal trend and its relationship with socioeconomic development and health coverage in China, Japan, and South Korea. In this way, we sought to inform the development of related policies and the allocation of health resources.

## METHODS

### Case definition and data sources

The GBD 2021 study defined anaemia as the decrease in haemoglobin concentrations, regardless of the morphology or cell function of red blood cells [13]. The thresholds of haemoglobin concentrations for the diagnosis of anaemia and assessment of severity (mild, moderate, and severe) varied by sex, age, and pregnancy status (Table S1 in the **Online Supplementary Document**), and GBD 2021 used criteria mainly based on previous WHO recommendations [15].

GBD 2021 used 731 representative data sources to obtain global epidemiological data on anaemia, including population surveys, published studies, and government reports, which were all summarised on the website of the Global Health Data Exchange (GHDx). Specific estimations for China were mainly based on 11 data sources, those for Japan on four data sources, and those for South Korea on eight data sources, including data on mean haemoglobin concentration and anaemia prevalence retrieved from the WHO Vitamin and Mineral Nutrition Information System [16]. Since haemoglobin concentration increases with altitude, raw data were adjusted for altitude using the formula recommended by the WHO [15], without additional adjustments were made for smoking status, method of haemoglobin sampling, or analysis method.

The GBD data we used here are publicly available on the GHDx website [17]. We extracted the HDI data of the world, China, Japan, and South Korea from 1990 to 2021 from the Human Development Report 2021/2022 [18]; indicators of populations in the world, China, Japan, and South Korea and their temporal trends from 1990 to 2021 from the World Population Prospects 2022 [19]; and the UHC data from 2000 to 2021 from the Global Health Observatory database [20]. We reported our findings per the Guidelines for Accurate and Transparent Health Estimates Reporting (GATHER) statement [21].

### Estimation of the prevalence and YLDs caused by anaemia

The estimation of the overall prevalence of anaemia was done in four steps [13]: through spatiotemporal Gaussian process regression models of the mean haemoglobin concentration; by calculating the ensemble weights; by generating the ensemble distributions; and by calculating the anaemia prevalence based on the ensemble distributions.

The YLD metric allows for standardised comparisons of non-fatal health burdens between diseases. YLDs for anaemia were calculated as the prevalence of severity-specific anaemia multiplied by the associated severity-specific disability weights, which represent the level of health loss associated with a given disease state, ranging between 0 (no health loss) and 1 (death) (Table S1 in the **Online Supplementary Document**) [13].

Uncertainty was propagated by sampling 1000 draws from the posterior distribution of each estimated metric using Monte Carlo simulation techniques. The 95% uncertainty intervals (UIs) for each metric, at each level of aggregation, were presented as the 2.5th and 97.5th percentile of the draws.

### Estimation of anaemia causal distribution

Anaemia can arise due to many different diseases. In GBD 2021, each case of anaemia was assigned to a single cause from 37 underlying mutually exclusive, collectively exhaustive causes [13]. Here we reclassified these causes into nine categories: chronic kidney disease (CKD); dietary iron deficiency; digestive system diseases; endocrine, metabolic, blood, and immune disorders; gynaecological diseases; hemoglobinopathies; infectious diseases; maternal haemorrhage; and vitamin A deficiency. Multiplying cause-specific haemoglobin shifts (which represented the associated mean difference in haemoglobin concentration) by the prevalence of that cause, could return a prevalence-weighted shift in the haemoglobin distribution specific to each cause, location, year, age, and sex, as detailed elsewhere [13].

### Statistical analysis

We used age-standardised rates (ASRs) of prevalence and YLDs to avoid the influences of population size and age composition. We calculated them as:



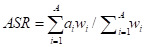


where *A* indicates the number of age groups in the reference standard population, while *a_i_* and *w_i_* indicate the age-specific rate and number of people in age group *i*.

We used joinpoint regression models to quantify temporal changes in ASRs of prevalence and YLDs due to anaemia [22]. We performed logarithmic transformations of the ASRs. Following a previous study [23], we calculated the standard errors of the GBD estimates as the corresponding width of 95% UI divided by 2 × 1.96 to fit the segmented log-linear model, *ln ASR = β*_0_ *+ β_i_* *× year + ε*. We calculated the annual percentage change (APC), indicating the annual change rate in specific segment, as *APC* = (*e^βᵢ^* − 1) *×* 100, and the average annual percentage change (AAPC), indicating the summarised APC in the whole study period, as *AAPC* = (*e ^∑ βᵢwᵢ/ ∑ wᵢ^* − 1) *×* 100. Here, *β_i_* indicates the slope coefficients of each segment in the expected year range, while *w_i_* is the year length of each segment in the year range. We performed the joinpoint analysis in the Joinpoint Regression Program, version 5.0.2 (National Cancer Institute, Bethesda, Maryland, USA).

We used Spearman’s correlation test to explore the relationship between the anaemia burden and the HDI or the UHC index. The HDI, developed by the United Nations Development Programme, is a summary measure to assess the average achievement in key dimensions of human development for a specific country or region. It is calculated as the geometric mean of the normalised indices for life expectancy at birth (health dimension), mean of years of schooling for adults aged 25 years (education dimension), and the logarithm of income (living standard dimension) measured by Gross National Income (GNI) per capita with the 2017 purchasing power parity in dollars. The UHC implies that all people can obtain the health services they need; as part of the Sustainable Development Goal (SDG) 3.8, it is measured by the SDG indicator 3.8.1, which is defined as the average coverage of essential services based on tracer interventions that include reproductive, maternal, newborn and child health; infectious diseases; non-communicable diseases; and service capacity and access. This indicator is measured as an index reported on a unitless scale of 0 to 100, which is computed as the geometric mean of 14 tracer indicators of health service coverage. We used the HDI and UCH indicators for the world, China, Japan, and South Korea to determine the influence of social development and health service coverage on the disease burden of anaemia. We conducted the correlation analyses in R, version 4.3.2 (R Core Team, Vienna, Austria). Statistical significance was set as a two-sided *P*-value <0.05.

## RESULTS

### The overall prevalence and YLDs due to anaemia in China, Japan, and South Korea

An estimated 136 million people (95% UI = 131, 141) among the 1.43 billion in China suffered from anaemia in 2021, amounting to a decrease of 37% compared to 1990. The age-standardised prevalence rate was 8.9% (95% UI = 8.6, 9.3). In 2021, anaemia caused 3.0 million (95% UI = 2.0, 4.4) YLDs in China, 44.3% lower than that in 1990; the ASR of YLDs was 200.8 per 100 000 person-years (95% UI = 130.8, 295.1), 59.3% lower than that in 1990 (**Table 1**, Figure S1 in the **Online Supplementary Document**).

**Table 1:** Comparison of anaemia burden in 2021 with that in 1990 specified by severity

Severity	Prevalent cases in 2021. thousands (95% UI)	Percentage change in cases from 1990	ASRs of prevalence in 2021, % (95% UI)	Percentage change in ASRs of prevalence from 1990	Total YLDs in 2021, thousands (95% UI)	Percentage change in total YLDs from 1990	ASRs of YLDs in 2021, per 100 000 population (95% UI)	Percentage change in ASRs of YLDs from 1990
World								
*Total*	1 919 910 (1 890 922, 1 951 845)	27.6	24.8 (24.4, 25.2)	−11.3	52 050 (34 779, 75 476)	11.5	680.7 (454.7, 988)	−20.5
*Mild*	1 114 455 (1 089 361, 1 141 127)	40.9	14.2 (13.9, 14.6)	−4.7	4086 (1461, 8993)	40.8	52.2 (18.6, 114.8)	−4.7
*Moderate*	731 496 (717 720, 744 456)	15.4	9.6 (9.4, 9.8)	−16.9	37 233 (24 536, 54 782)	15.3	488.4 (321.8, 719.5)	−16.9
*Severe*	73959 (70 690, 77 627)	−6.7	1 (0.9, 1)	−34.7	10 731 (7324, 15 130)	−6.8	140 (95.6, 197.4)	−34.7
China								
*Total*	136 263 (131 223, 141 786)	−37	8.9 (8.6, 9.3)	−53.1	3016 (1964, 4440)	−44.3	200.8 (130.8, 295.1)	−59.3
*Mild*	90 274 (86 768, 94 249)	−32.7	5.8 (5.6, 6.1)	-49.5	331 (119, 719)	−32.9	21.4 (7.7, 46.5)	−49.6
*Moderate*	42 331 (40 487, 44 184)	−43.1	2.8 (2.7, 3)	−57.6	2152 (1409, 3172)	−43.4	145.2 (94.3, 213.4)	−57.6
*Severe*	3659 (3408, 3911)	−52.2	0.2 (0.2, 0.2)	−68.7	533 (363, 741)	−52.4	34.3 (23.3, 47.8)	−68.6
Japan								
*Total*	13 564 (11 785, 16 021)	8.9	7.4 (6.1, 9)	−22.6	181 (108, 282)	39.1	79.2 (44.7, 129.9)	−21.2
*Mild*	11 135 (9475, 13 226)	3.4	6.3 (5.1, 7.9)	−22.9	41 (15, 92)	3	23.3 (8.6, 54.4)	−22.8
*Moderate*	2230 (1883, 2633)	35.8	1 (0.8, 1.2)	−21.1	113 (70, 169)	34.5	50.6 (31.6, 80.1)	−20.9
*Severe*	200 (111, 323)	297.8	0 (0, 0.1)	−17.3	28 (13, 50)	288.7	5.3 (2.8, 9.4)	−16.8
South Korea								
*Total*	2673 (2363, 2983)	−36.6	4.7 (4.1, 5.3)	−55	34 (22, 55)	−25.7	60.4 (38.2, 94.7)	−50
*Mild*	2184 (1919, 2461)	−39.2	3.8 (3.3, 4.3)	−56.3	8 (3, 18)	−39.3	14 (5.1, 30.3)	−56.3
*Moderate*	474 (388, 559)	-23	0.8 (0.7, 1)	−48.2	24 (16, 37)	−23.3	42.8 (28.3, 65.5)	−48.2
*Severe*	16 (9, 25)	40.5	0 (0, 0)	−42.6	2 (1, 4)	39.7	3.6 (1.8, 6.3)	−41.7

ASR – age-standardised rate, UI – uncertainty interval, YLDs – years lived with disability

Among the 125 million population in Japan, there were 13.6 million (95% UI = 11.8, 16.0) anaemia cases in 2021, 8.9% more than in 1990. The ASR of prevalence was 7.4% (95% UI = 6.1, 9.0), 22.6% lower than that in 1990. In 2021, anaemia accounted for 181 thousand YLDs (95% UI = 108, 282) in Japan, 39.1% higher than that in 1990. The ASR of YLD was 79.2 per 100 000 person-years (95% UI = 44.7, 129.9), 21.2% lower than that in 1990 (**Table 1**, Figure S1 in the **Online Supplementary Document**).

In South Korea, there were 2.7 million (95% UI = 2.4, 3.0) anaemia cases in 2021 among the 52 million population, presenting a decrease of 36.6% compared to 1990. The ASR of prevalence was 4.7% (95% UI = 4.1, 5.3), 55.0% lower than that in 1990. In 2021, anaemia accounted for 34 thousand YLDs (95% UI = 22, 55) in South Korea. Here, the ASR of YLD was 60.4 per 100 000 person-years (95% UI = 38.2, 94.7), 50.0% lower than that in 1990 (**Table 1**, Figure S1 in the **Online Supplementary Document**).

**Figure 1:**
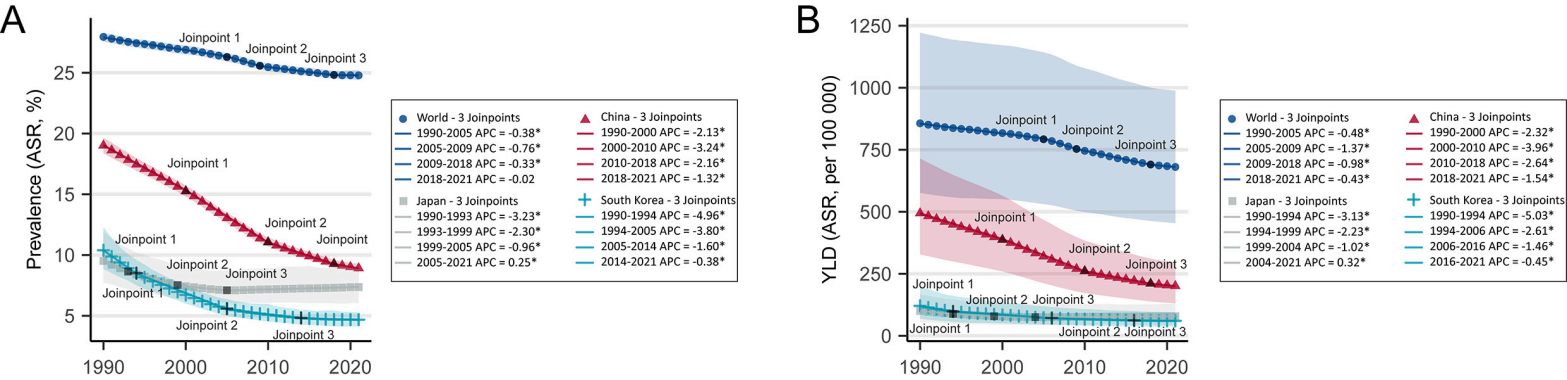
Joinpoint analysis of the overall anaemia burden in the world, China, Japan, and South Korea from 1990 to 2021. **Panel A.** Trends of ASRs of anaemia prevalence rates at the global level, China, Japan, and South Korea. **Panel B.** Trends of ASRs of YLDs caused by anaemia in the world, China, Japan, and South Korea. ASR – age-standardised rate, YLDs – years lived with disability.

For the temporal trend of anaemia prevalence and YLD, their ASRs declined gradually each year at the global level. In China, the slopes were steeper for the whole range; in Japan, they flattened after 1999 (APC<2%), which also occurred in South Korea around 2005 (**Figure 1**, Table S2–3 in the **Online Supplementary Document**).

### The gender discrepancy of anaemia burden in China, Japan, and South Korea, compared with the world

**Figure 2:**
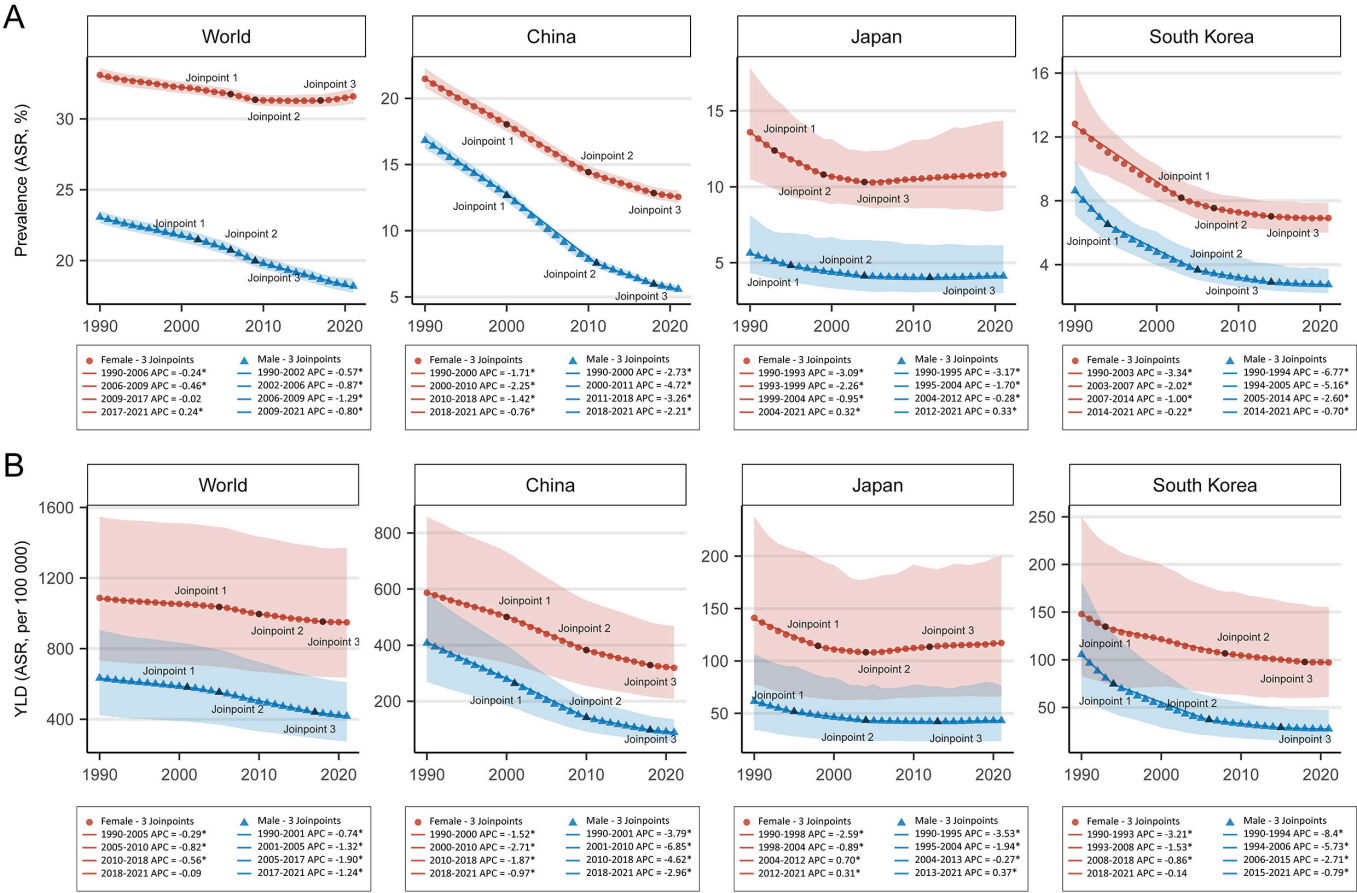
Joinpoint analysis of sex-specific anaemia burden at the global level, and in China, Japan, and South Korea from 1990 to 2021. **Panel A.** Trends of ASRs of anaemia prevalence rate at the global level, and in China, Japan, and South Korea. **Panel B.** Trends of ASRs of YLDs caused by anaemia at the global level, and in China, Japan, and South Korea. ASR – age-standardised rates, YLDs – years lived with disability.

In China, the prevalence of anaemia was almost twice higher in women than in men in 2021, while the gender discrepancy in YLDs caused by anaemia was even larger. Regarding the temporal trend of sex-specific anaemia burden, we observed more significant reductions in men than in women in all periods from 1990 to 2021. The gender discrepancy of anaemia burden was similar, but larger in South Korea, where anaemia caused over three times more YLDs in women than in men in 2021, a reduction of >50% was found since 1990, also much higher than that in women. The gender discrepancy of anaemia burden in Japan was similar, but not that prominent; concerning the dynamic changes, the temporal trends appeared to be similarly stable since 2004 in both sexes (**Figure 2****,** **Table 2****,** Figure S2 in the **Online Supplementary Document**).

**Table 2:** Comparison of anaemia burden in 2021 with that in 1990 stratified by sex

Sex	Prevalent cases in 2021, thousands (95% UI)	Cases change from 1990 in %	ASR of prevalence in 2021, % (95% UI)	Percentage change in ASRs of prevalence from 1990	Total YLDs in 2021, thousands (95% UI)	Percentage change in total YLDs from 1990	ASR of YLDS in 2021, per 100 000 person-years (95% UI)	Percentage change in ASRs of YLDs from 1990
World								
*Women*	1 227 504 (1 207 955, 1 248 263)	38.7	31.6 (31.1, 32.1)	−4.6	36 605 (24 537, 52 860)	25.4	949 (635.8, 1370.9)	−12.7
*Men*	692 406 (674 422, 712 152)	11.9	18.2 (17.7, 18.7)	−21.1	15 445 (10 157, 22 654)	−11.8	417.1 (274.4, 609)	−34.1
China								
*Women*	94 194 (90 839, 97 654)	−23.7	12.6 (12.1, 13)	−41.5	2415 (1574, 3539)	−26.3	319.4 (207.5, 467.8)	−45.6
*Men*	42 069 (39 806, 44 408)	−54.7	5.6 (5.3, 5.9)	−66.8	601 (385, 935)	−71.9	88.8 (57, 135.4)	−78.2
Japan								
*Women*	8986 (7367, 11224)	−2.8	10.8 (8.5, 14.3)	−20.4	126 (75, 196)	29	117.2 (66.1, 199.6)	−16.9
*Men*	4578 (3491, 6195)	42.6	4.1 (3, 6.2)	−26.8	55 (31, 91)	69.2	43.1 (23.6, 76.6)	−30
South Korea								
*Women*	1908 (1659, 2164)	−32.6	6.9 (6, 7.9)	−46	27 (17, 44)	−13.5	97.2 (61.1, 154.9)	−34.3
*Men*	765 (620, 947)	−44.8	2.7 (2.2, 3.7)	−68.2	7 (4, 12)	−51.1	27.4 (15, 47.8)	−74

ASR – age-standardised rate, UI – uncertainty interval, YLDs – years lived with disability

### The age distribution of anaemia burden in China, Japan, and South Korea, compared with the world

**Figure 3:**
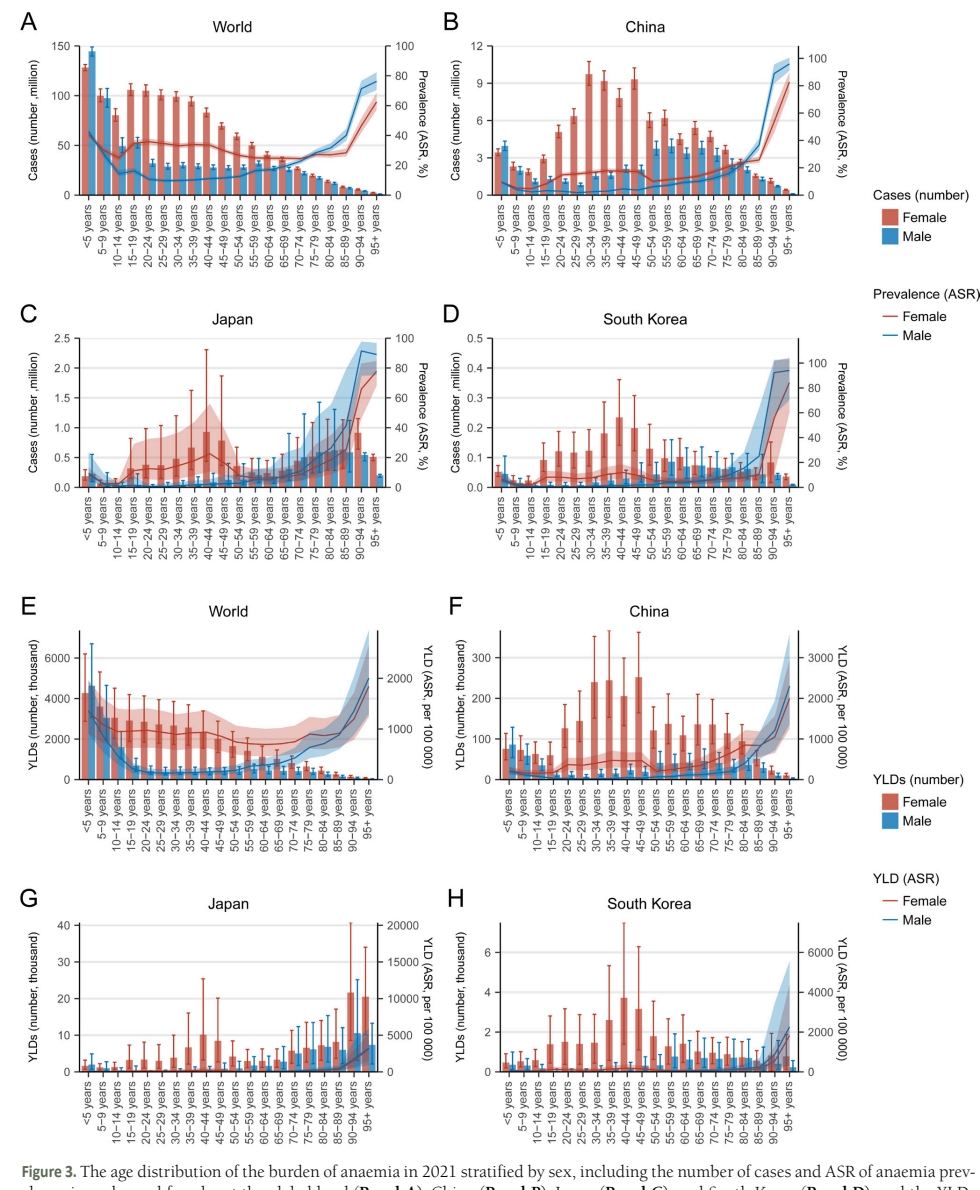
The age distribution of the burden of anaemia in 2021 stratified by sex, including the number of cases and ASR of anaemia prevalence in males and females at the global level (**Panel A**), China (**Panel B**), Japan (**Panel C**), and South Korea (**Panel D**), and the YLDs and ASRs of YLD caused by anaemia in males and females at the global level (**Panel E**), China (**Panel F**), Japan (**Panel G**), and South Korea (**Panel H**).

At the global level in 2021, children aged two to nine years old still accounted for the highest number of anaemia cases and YLDs. In comparison, the highest numbers of anaemia cases and YLDs in China and South Korea occurred in young and middle-aged adults, especially in women, while old adults also bore a major part of the anaemia burden in Japan (**Figure 3**).

**Figure 4:**
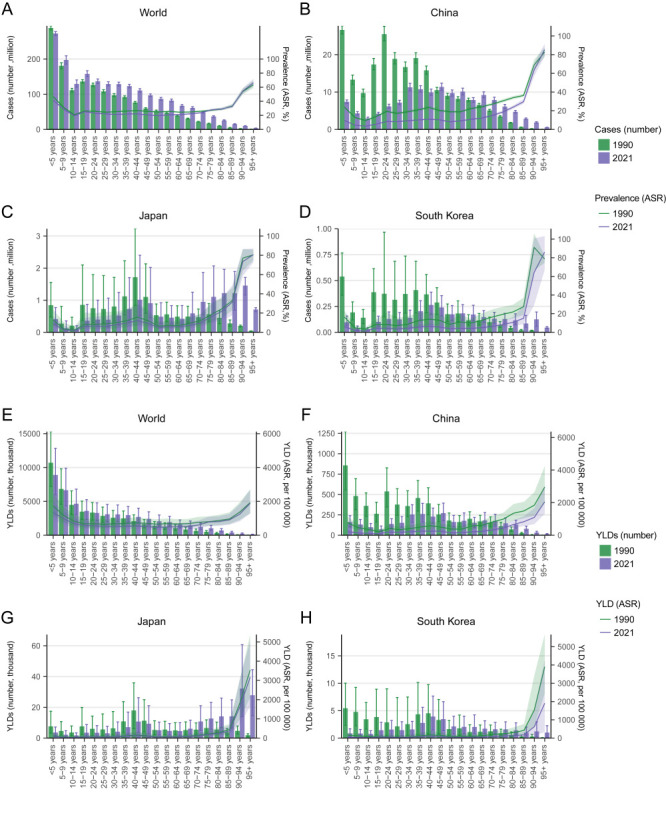
The age distribution of the burden of anaemia in 2021 compared with that in 1990 including the age-specific number of cases and ASRs of anaemia prevalence at the global level (**Panel A**), China (**Panel B**), Japan (**Panel C**), and South Korea (**Panel D**), and the age-specific YLDs and ASRs of YLD caused by anaemia at the global level (**Panel E**), China (**Panel F**), Japan (**Panel G**), and South Korea (**Panel H**).

Compared with 1990, all three countries saw a reduction of the anaemia burden in children and young adults, particularly China and South Korea; meanwhile, anaemia cases and YLDs in old adults increased in all three countries, but especially in Japan (**Figure 4**).

### The cause distribution of anaemia burden in China, Japan, and South Korea

**Figure 5:**
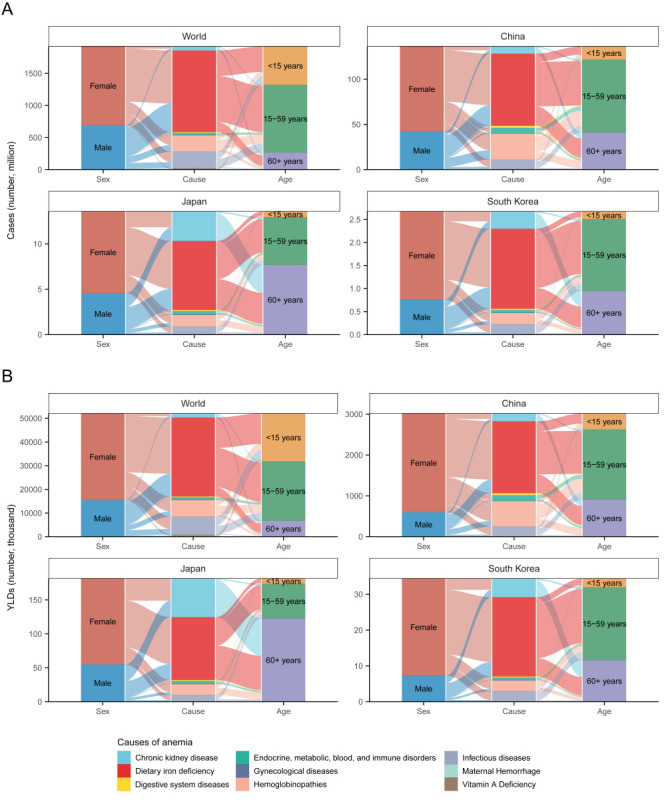
The proportions of cause-specific anaemia and sex and age distribution of cause-specific anaemia in 2021. **Panel A.** Prevalent cases of cause-specific anaemia. **Panel B.** YLDs due to cause-specific anaemia.

**Figure 6:**
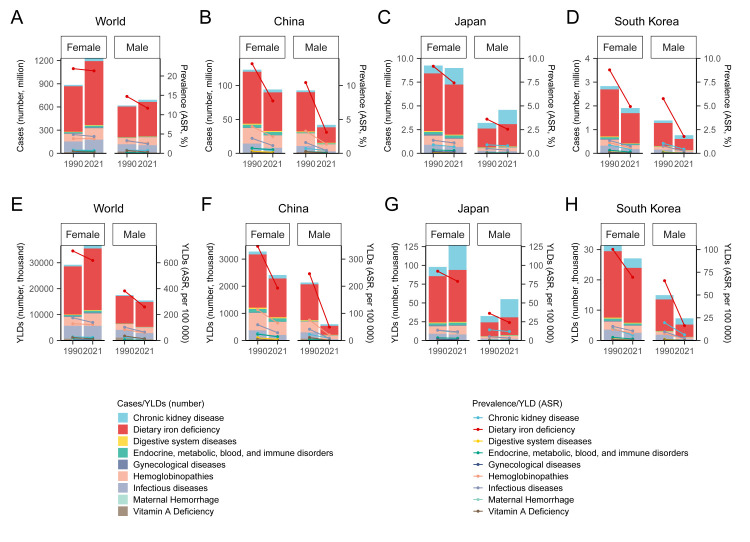
The changes in cause-specific burden of anaemia from 1990 to 2021, including the cause-specific number of cases and ASRs of anaemia prevalence at the global level (**Panel A**), China (**Panel B**), Japan (**Panel C**), and South Korea (**Panel D**), and the cause-specific YLDs and ASRs of YLD due to anaemia at the global level (**Panel E**), China (**Panel F**), Japan (**Panel G**), and South Korea (**Panel H**).

In 2021, dietary iron deficiency remained the leading cause of anaemia across all three countries in both men and women, although the ASRs of prevalence and YLDs have been reducing since 1990, which was consistent with the global data. Noticeably, haemoglobinopathies were the second contributor to anaemia cases and YLDs in China in both men and women. Meanwhile, CKD ranked second in cause-specific anaemia cases and YLDs in Japan and South Korea; both their numbers have been increasing, especially in old adults. The proportion of anaemia cases due to infectious disease decreased in all three countries, but remained noticeable in its absolute amounts, especially in China (**Figure 5**, **Figure 6**).

### The association of anaemia burden with social development in China, Japan, and South Korea, compared with the world

**Figure 7:**
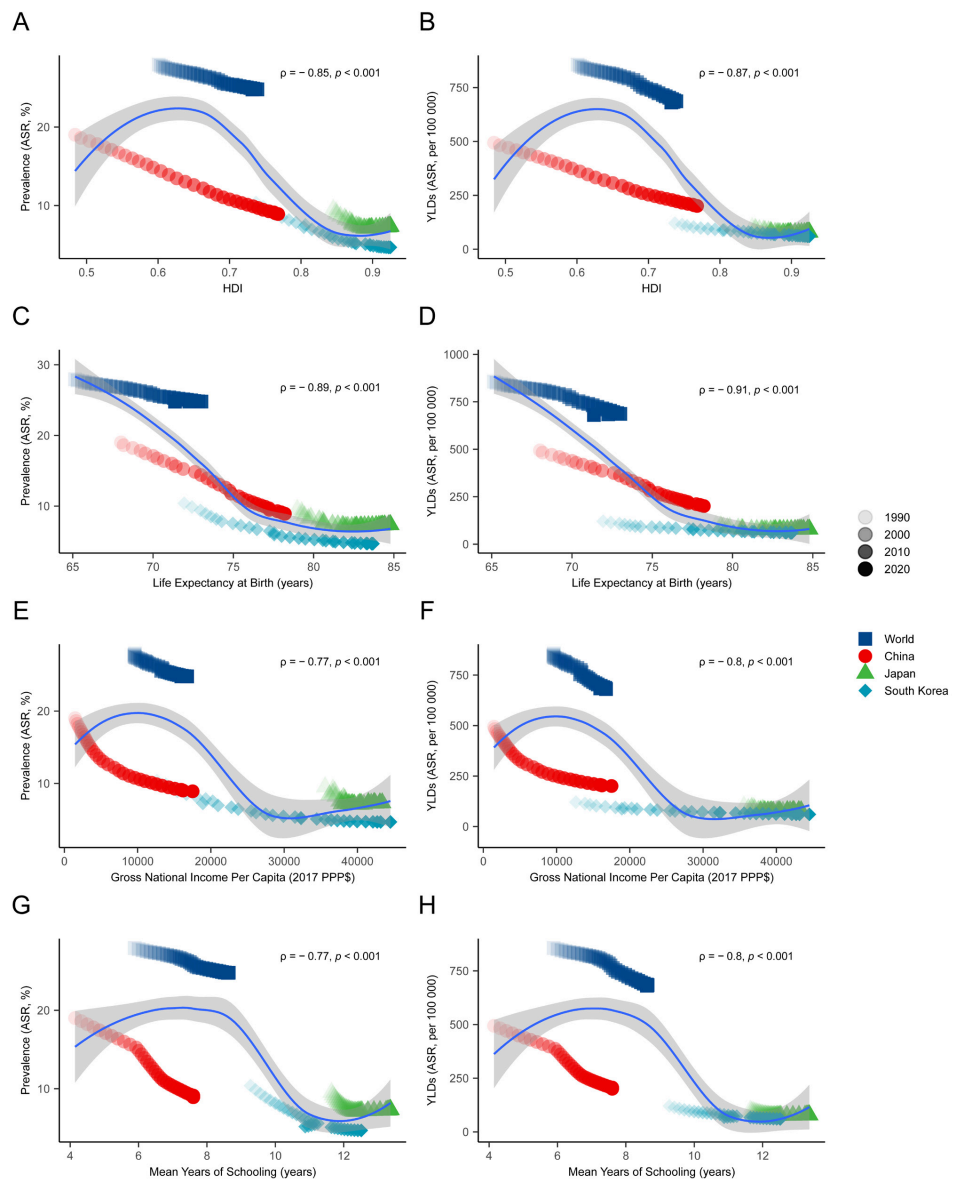
The correlation of the burden of anaemia with social development. **Panel A, Panel B.** Correlations of ASRs of anaemia prevalence and YLD of anaemia with the HDI. **Panel C, Panel D.** Correlation with life expectancy at birth. **Panel E, Panel F.** Correlation with the GNI per capita. **Panel G, Panel H.** Correlation with average years of education.

The anaemia burden generally tended to decrease along with socioeconomic development, whereby countries with higher HDI levels had lower anaemia prevalence and YLD rates. We observed a negative association between anaemia burden and HDI in cases where HDI≤0.85, which was mainly seen in the world and China. This association was weakened and possibly reversed when HDI>0.85, which we observed in Japan and South Korea. From the point of HDI components, we found that the anaemia burden was negatively associated with a life expectancy at birth of no more than 80 years; with per-capita income when the GNI per capita was no more than USD 20 000; and with education level when mean years of schooling were no more than nine years (**Figure 7**).

### The association of anaemia burden with health service coverage in China, Japan, and South Korea, compared with the world

**Figure 8:**
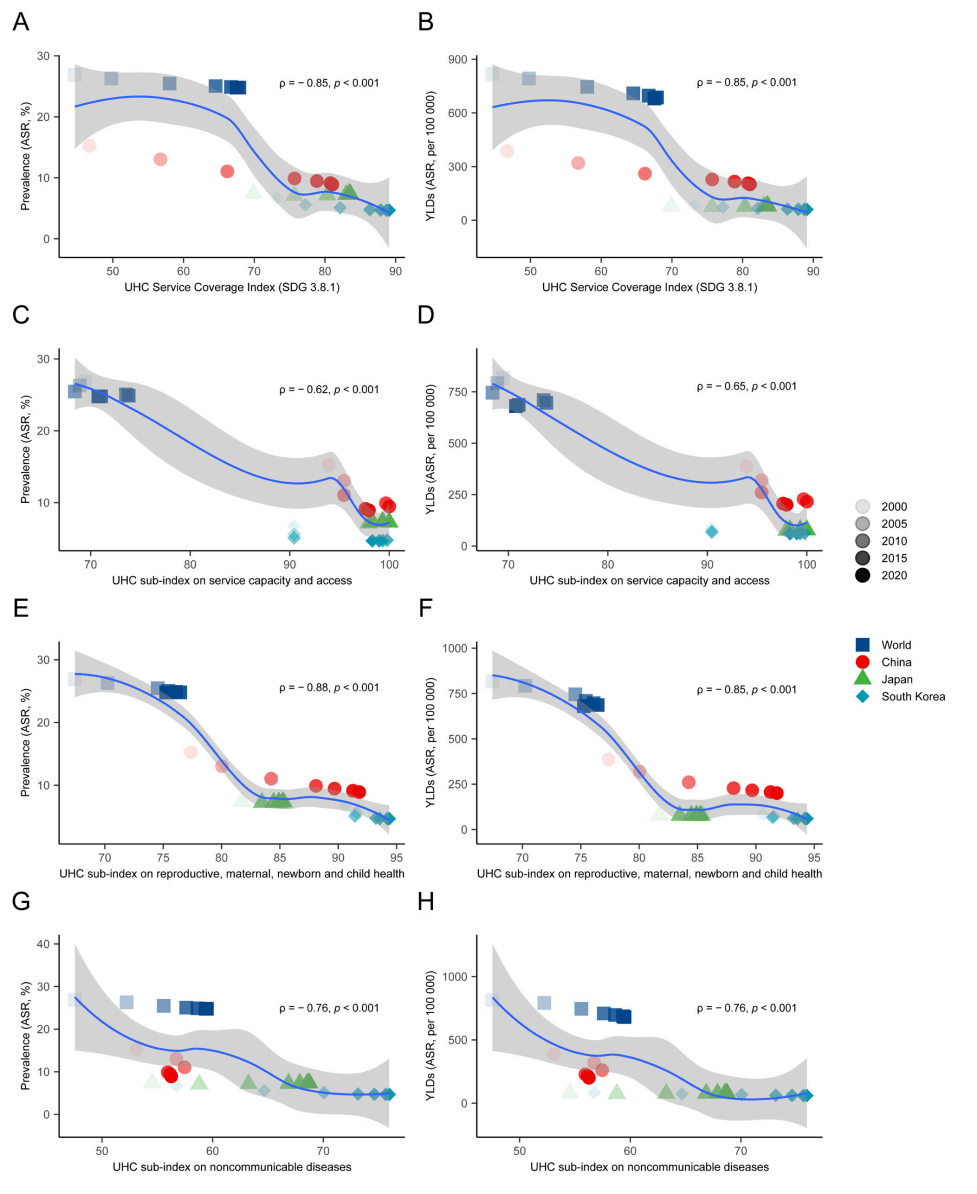
The correlation of the burden of anaemia with the UHC index. **Panel A, Panel B.** Correlation of ASRs of anaemia prevalence and YLDs of anaemia with the UHC index (SDG 3.8.1). **Panel C, Panel D.** Correlation with the UHC sub-index on service capacity and access. **Panel E, Panel F.** Correlation with the UHC sub-index on reproductive, maternal, newborn, and child health. **Panel G, Panel H.** Correlation with the UHC sub-index on noncommunicable diseases.

We found similar negative associations between the anaemia burden and health service coverage, in which the ASRs of anaemia prevalence and YLDs tended to decrease in populations that were better covered with essential health services. Concerning different aspects of health services coverage, we saw a stronger correlation between the anaemia burden (rho = −0.88) and the UHC sub-index (rho = −0.85) with the reproductive, maternal, newborn, and child health in these countries, possibly due to women of reproductive age and children being a high-risk population for anaemia (**Figure 8**).

## DISCUSSION

As evidenced in data from the GBD 2021 study, anaemia still affected many individuals in China, Japan, and South Korea, which consequently accounted for substantial YLDs in these countries. From 1990 to 2021, the rates of prevalence and YLDs caused by anaemia have been reducing gradually; however, the slopes have been flattened in recent years, especially in Japan and South Korea. The gender gap in the anaemia burden was still rather prominent in the three countries, and was greater than the global average, especially in adults of reproductive age. The peak of the age distribution of anaemia burden shifted toward higher age groups in all three countries, especially in Japan. Although dietary iron deficiency was still the leading cause in all age groups collectively, CKD was becoming responsible for a growing share of anaemia cases and YLDs in all three countries, especially in adults aged more than 60 years in Japan and South Korea. Meanwhile, haemoglobinopathies were another noticeable cause of anaemia in China. Due to differences in the initial socioeconomic levels of the three countries, the role of social development in decreasing the burden of anaemia showed discrepant patterns in the past three decades. However, improvements in essential health service coverage had positive effects in decreasing the burden of anaemia.

Some studies, mainly by the GBD Anemia Collaborators, have previously investigated the global burden of anaemia [13,24-26]. They found significant disparities in the burden due to anaemia at the country level, with prevalence in western sub-Saharan countries being more than 10 times higher than that in northern European countries [13]. The burden of anaemia in the three East Asian countries was moderated when analysed at the global level, but it was rarely reported collectively at the national level. Due to their large populations, the inevitable population ageing, and the decelerating economic growth, further reducing the burden of anaemia remains challenging for all three countries.

In line with the global trend, the burden of anaemia in China and South Korea declined significantly in the past thirty years, primarily due to better access to health care services, improvements in diet and nutrition, infectious disease control, and effective treatment of anaemia [26]. The absolute numbers of prevalent cases and YLDs caused by anaemia in Japan showed an initially decreasing, followed by a rising trend in the same period, in which the effect of social development was probably overwhelmed by that of the ageing of its population [27] (Figure S3 in the **Online Supplementary Document**).

We observed a significant gender gap in the burden of anaemia in these three East Asian countries, which was mainly due to some women-specific pathophysiological characteristics, such as regular blood loss resulting from menstrual bleeding, pregnancy-related complications, and abnormal uterine bleeding. More notably, health-related gender inequalities are very prominent in East Asian countries [28], where women were more likely to be engaged in childcare and other household affairs [29] and less likely to receive adequate medical care [30,31] and health insurance coverage [32]. Comprehensive social interventions are needed to reduce the gender gap of the burden of anaemia, including more education for girls and women [33], decreasing occupational segregation [34], timely treatment of abnormal uterine bleeding [35], as well as use of contraceptives and access to iron-fortified foods [36,37].

Anaemia in infants and preschool children has always been a priority of national child health care, but its prevalence is still high, especially in less developed regions of China [38,38]. Adherence to the home fortification programme with micronutrient supplementation has proven to be effective against anaemia in children [40–42]. The burden of anaemia on young and middle-aged adults in the three countries was primarily borne by women of reproductive age, as discussed above. Compared with the circumstances in 1990, the peaks of anaemia burden shifted consistently to aged adults in all three countries, which was most prominent in Japan. In view of the age structure of the population, it was estimated that adults over 65 constituted 27.7% of Japan’s total population in 2017 which is the highest in the world, which was predicted to increase to 38.4% in 2065 [27] (Figure S3 in the **Online Supplementary Document**). In practice, population ageing is the result of rapid economic growth and the development of health care, where Japan remains in the lead, China is predicted to have more rapid population ageing in the next 25 years [43]. This suggest that anaemia in the elderly is and will be a shared health concern for all three countries in the future [44,45]. The aetiology of anaemia in the elderly is more complicated and usually multifactorial, as it includes nutrition deficiency, inflammation caused by CKD, infectious diseases and tumours, and unexplained cases; therefore, physicians should personalise treatment to each individual [9,46].

In line with global trends, dietary iron deficiency was still the primary cause of anaemia in the three East Asian countries in both men and women. It was previously proven to be associated with serious adverse outcomes, especially in infants [47], as well as pregnant and non-pregnant women [48,49]. Delayed cord clamping until 1–3 minutes after birth is a simple, but effective intervention to reduce newborn anaemia by facilitating placental transfusion and iron-rich blood flow to the newborn [47,50], while routine iron supplementation is recommended for high-risk infants 6 to 12 months of age [51]. Moreover, preventive iron supplementation is recommended for all pregnant women, non-pregnant adult women, adolescent girls, and perimenopausal women with a high prevalence of anaemia [52]. A recent meta-analysis found that consuming condiments and seasonings fortified with iron plus other micronutrients may prevent anaemia in the general population [53]. In China, haemoglobinopathies accounted for ~ 20% of all anaemia cases, ranking second in all cause-specific anaemia. Specifically, thalassemia, a monogenic recessive haematological disease, caused the most significant proportion of this burden; it was previously found to affect approximately 10% population in some southern provinces of China [54]. Currently, some progress has been made in genetic diagnosis [55] and antenatal screening for thalassemia [56,57]. In Japan and South Korea, CKD accounted for a considerable share of anaemia cases and YLDs, especially in the aged population. In Japan, anaemia affects approximately 32% of patients with stage 3–5 CKD [58]. Apart from conventional treatment, chronic renal anaemia may involve the use of recombinant human erythropotein and its derivatives [59,60], which still need further studies to verify their effectiveness.

Considering the role of social development, gross negative associations were observed between anaemia burden and HDI in the three East Asian countries, especially in China [13]. Low HDIs indicated lower levels of income and education, which were linked to poor nutritional status, inadequate access to health services, and macro-level gender inequality, thereby resulting in a high prevalence of anaemia, especially in children and women of reproductive age [61-63]. As a developing country, China could still benefit from socioeconomic development in reducing the anaemia burden, the effect of which was not that obvious in highly developed Japan and South Korea.

This study had several strengths. To our knowledge, it is the first to simultaneously explore the prevalence and YLDs due to anaemia in China, Japan, and South Korea, stratified by sex, age, and causes. It also provides the most up-to-date estimations from the GBD 2021, which comprised comprehensive and systematic data sets for the disease burden at national and regional levels.

However, the limitations of the study should also be noted. First, although the GBD 2021 study synthesised many input sources to guarantee accuracy, deviations from true, real-life circumstances are inevitable, as the data were fitted by mathematic models. Second, due to the scarcity of data sources for specific populations, such as infants younger than six months, adult men, and adults older than 60 years, the uncertainty of corresponding estimations was relatively large. This may be improved by strengthening health data collecting and sharing in the future. Third, each anaemia case was assigned with a single cause in GBD 2021; however, some cases were more likely to be induced by multiple factors, meaning that some cause-specific estimations could be underestimated to some extent. Fourthly, many conditions could result in anaemia, but for those less prevalent or with sparse data, they may be neglected or be combined with other causes; this warrants further investigations when focussing on these causes. Finally, there was still large heterogeneity in population structure and socioeconomic level for different parts of each country, as was the case for China. Additional sub-national analyses could further inform context-appropriate policies.

## CONCLUSIONS

Anaemia remains a major public health concern in China, Japan, and South Korea, although some progress has been achieved in the past three decades, mainly in children and young adults. To reduce this burden effectively and efficiently, high-risk populations should be targeted for anaemia surveillance and intervention, including young children and women of reproductive age, but also the elderly, considering that population ageing is inevitable. Chronic renal anaemia may require more attention, especially for the elderly in developed societies, despite dietary iron deficiency remaining the leading cause of most anaemia cases.

## Additional material


Online Supplementary Document.
