# Supplementary material for: The temporal trends of prevalence and years lived with disability of anaemia in China, Japan, and South Korea, from 1990 to 2021: Results from the Global Burden of Disease Study 2021
Source: J Glob Health. 2024 May 24;14:04073. doi: 10.7189/jogh.14.04073 (PMC11112532; doi:10.7189/jogh.14.04073)
Supplement: Online Supplementary Document. [file jogh-14-04073-s001.pdf]

Tables

Supplementary file 1 Table S1 Definitions of anemia severity and corresponding disability weights in GBD 2021.

Supplementary file 2 Table S2 Joinpoint analysis of age-standardized rates of anemia prevalence from 1990 to 2021.

Supplementary file 3 Table S3 Joinpoint analysis of age-standardized rates of YLD caused by anemia from 1990 to 2021.

Figures

Supplementary file 4 Figure S1 The overall anemia burden in the world, China, Japan, and South Korea from 1990 to 2021, stratified by severity. A. Prevalent anemia cases in the world, China, Japan, and South Korea from 1990 to 2021; B. Age-standardized rates of anemia prevalence in the world, China, Japan, and South Korea from 1990 to 2021; C. YLDs due to anemia in the world, China, Japan, and South Korea from 1990 to 2021; D. Age-standardized rates of YLD due to anemia in the world, China, Japan, and South Korea from 1990 to 2021.

Supplementary file 5 Figure S2 The sex-specific anemia burden in the world, China, Japan, and South Korea from 1990 to 2021. A. Prevalent anemia cases in males and females in the world, China, Japan, and South Korea from 1990 to 2021; B. Age-standardized rates of anemia prevalence in males and females in the world, China, Japan, and South Korea from 1990 to 2021; C. YLDs due to anemia in males and females in the world, China, Japan, and South Korea from 1990 to 2021; D. Age-standardized rates of YLD due to anemia in males and females in the world, China, Japan, and South Korea from 1990 to 2021.

Supplementary file 6 Figure S3 The trend of the total population (A) and age of populations structure (B) in the world, China,Japan, and South Korea from 1990 to 2021. The red line and black line indicate the numbers of birth and death in each year.

Table S1. Definitions of anemia severity and corresponding disability weights in GBD 2021.

	Mild	Moderate	Severe
Age 0-6 days			
Males/Females	145-159	100-144	<100
Age 7-27 days			
Males/Females	120-134	85-119	<85
Age 1 month- 4 years			
Males/Females	100-109	70-99	<70
Age 5-14 years			
Males/Females	110-114	70-99	<70
Age ≥ 15 years			
Males	110-129	80-109	<80
Females, non-pregnant	110-119	80-109	<80
Females, pregnant	100-109	70-99	<70
Disability weights (95% UI)	0.004 (0.001-0.008)	0.052 (0.034-0.076)	0.149 (0.101-0.209)

Table S2. Joinpoint analysis of age-standardized rate of prevalence.

	Sex	Segment	Period	APC/AAPC* (95% CI)	Test Statistic	P-Value
World	Both	0	1990-2005	-0.38(-0.39, -0.37)	-164.11	<0.001
		1	2005-2009	-0.76(-0.89, -0.63)	-209.01	<0.001
		2	2009-2018	-0.33(-0.36, -0.3)	-88.57	<0.001
		3	2018-2021	-0.02(-0.18, 0.14)	-13.2	<0.001
		**Full Range**	**1990-2021**	**-0.38(-0.41, -0.36)**	**-228.42**	**<0.001**
	Female	0	1990-2006	-0.24(-0.25, -0.24)	-257.44	<0.001
		1	2006-2009	-0.46(-0.66, -0.25)	-106.2	<0.001
		2	2009-2017	-0.02(-0.05, 0.01)	-13.85	<0.001
		3	2017-2021	0.24(0.16, 0.33)	-88.42	<0.001
		**Full Range**	**1990-2021**	**-0.15(-0.17, -0.12)**	**-137.84**	**<0.001**
	Male	0	1990-2002	-0.57(-0.59, -0.55)	-38.39	<0.001
		1	2002-2006	-0.87(-1.09, -0.66)	-8.37	<0.001
		2	2006-2009	-1.29(-1.72, -0.87)	-183.21	<0.001
		3	2009-2021	-0.8(-0.83, -0.78)	-234.75	<0.001
		**Full Range**	**1990-2021**	**-0.77(-0.82, -0.72)**	**-98.77**	**<0.001**
China	Both	0	1990-2000	-2.13(-2.16, -2.11)	-59.27	<0.001
		1	2000-2010	-3.24(-3.27, -3.21)	-104.19	<0.001
		2	2010-2018	-2.16(-2.21, -2.11)	-44.89	<0.001
		3	2018-2021	-1.32(-1.52, -1.11)	61.57	<0.001
		**Full Range**	**1990-2021**	**-2.42(-2.45, -2.39)**	**-13.95**	**<0.001**
	Female	0	1990-2000	-1.71(-1.73, -1.69)	-26.62	<0.001
		1	2000-2010	-2.25(-2.27, -2.23)	-9.23	<0.001
		2	2010-2018	-1.42(-1.45, -1.4)	23.36	<0.001
		3	2018-2021	-0.76(-0.87, -0.65)	-37.89	<0.001
		**Full Range**	**1990-2021**	**-1.72(-1.73, -1.7)**	**-36.28**	**<0.001**
	Male	0	1990-2000	-2.73(-2.79, -2.66)	-4.5	<0.001
		1	2000-2011	-4.72(-4.79, -4.65)	7.94	<0.001
		2	2011-2018	-3.26(-3.44, -3.09)	-99.4	<0.001
		3	2018-2021	-2.21(-2.76, -1.67)	-21.96	<0.001
		**Full Range**	**1990-2021**	**-3.51(-3.58, -3.44)**	**-35.57**	**<0.001**
Japan	Both	0	1990-1993	-3.23(-3.34, -3.12)	-43.75	<0.001
		1	1993-1999	-2.3(-2.34, -2.25)	-166.43	<0.001
		2	1999-2005	-0.96(-1, -0.92)	-45.7	<0.001
		3	2005-2021	0.25(0.24, 0.26)	-8.87	<0.001
		**Full Range**	**1990-2021**	**-0.82(-0.84, -0.81)**	**-136.39**	**<0.001**
	Female	0	1990-1993	-3.09(-3.54, -2.63)	-10.66	<0.001
		1	1993-1999	-2.26(-2.44, -2.09)	-15.4	<0.001
		2	1999-2004	-0.95(-1.16, -0.73)	-3.99	0.001
		3	2004-2021	0.32(0.29, 0.35)	-50.8	<0.001
		**Full Range**	**1990-2021**	**-0.72(-0.78, -0.66)**	**-140.76**	**<0.001**
	Male	0	1990-1995	-3.17(-3.34, -3)	-39.13	<0.001

		1	1995-2004	-1.7(-1.79, -1.6)	-8.17	<0.001
		2	2004-2012	-0.28(-0.41, -0.15)	-116.39	<0.001
		3	2012-2021	0.33(0.24, 0.41)	-59.39	<0.001
		**Full Range**	**1990-2021**	**-0.99(-1.04, -0.94)**	**-104.6**	**<0.001**
South Korea	Both	0	1990-1994	-4.96(-5.19, -4.73)	-69.19	<0.001
		1	1994-2005	-3.8(-3.85, -3.76)	-11.88	<0.001
		2	2005-2014	-1.6(-1.67, -1.53)	-23.14	<0.001
		3	2014-2021	-0.38(-0.47, -0.29)	-0.32	0.749
		**Full Range**	**1990-2021**	**-2.55(-2.6, -2.51)**	**-31.39**	**<0.001**
	Female	0	1990-2003	-3.34(-3.39, -3.29)	-60.37	<0.001
		1	2003-2007	-2.02(-2.41, -1.63)	-4.62	<0.001
		2	2007-2014	-1(-1.14, -0.87)	-1.63	0.117
		3	2014-2021	-0.22(-0.33, -0.11)	5.91	<0.001
		**Full Range**	**1990-2021**	**-1.94(-2.01, -1.88)**	**-12.41**	**<0.001**
	Male	0	1990-1994	-6.77(-7.04, -6.5)	-51.48	<0.001
		1	1994-2005	-5.16(-5.23, -5.08)	-8.54	<0.001
		2	2005-2014	-2.6(-2.74, -2.46)	-6.33	<0.001
		3	2014-2021	-0.7(-0.88, -0.52)	-60.71	<0.001
		**Full Range**	**1990-2021**	**-3.64(-3.71, -3.57)**	**-31.13**	**<0.001**

* APC (annual percentage change) for each period and AAPC (average annual percentage change) for the full range.

Table S3. Joinpoint analysis of age-standardized rate of YLDs.

	Sex	Segment	Period	APC/AAPC* (95% CI)	Test Statistic	P-Value
World	Both	0	1990-2005	-0.48(-0.5, -0.46)	-134.49	<0.001
		1	2005-2009	-1.37(-1.6, -1.15)	-193.73	<0.001
		2	2009-2018	-0.98(-1.02, -0.93)	-85.87	<0.001
		3	2018-2021	-0.43(-0.65, -0.2)	-13.24	<0.001
		**Full Range**	**1990-2021**	**-0.73(-0.77, -0.7)**	**-103.61**	**<0.001**
	Female	0	1990-2005	-0.29(-0.3, -0.27)	-159.75	<0.001
		1	2005-2010	-0.82(-0.95, -0.7)	-73.6	<0.001
		2	2010-2018	-0.56(-0.62, -0.51)	-10.02	<0.001
		3	2018-2021	-0.09(-0.29, 0.11)	-101.69	<0.001
		**Full Range**	**1990-2021**	**-0.43(-0.46, -0.4)**	**-111.37**	**<0.001**
	Male	0	1990-2001	-0.74(-0.78, -0.69)	-56.75	<0.001
		1	2001-2005	-1.32(-1.65, -0.98)	-9.36	<0.001
		2	2005-2017	-1.9(-1.95, -1.86)	-176.55	<0.001
		3	2017-2021	-1.24(-1.46, -1.02)	-144.1	<0.001
		**Full Range**	**1990-2021**	**-1.33(-1.38, -1.28)**	**-111.86**	**<0.001**
China	Both	0	1990-2000	-2.32(-2.36, -2.29)	-33.56	<0.001
		1	2000-2010	-3.96(-4, -3.92)	-23.92	<0.001
		2	2010-2018	-2.64(-2.7, -2.58)	-10.55	<0.001
		3	2018-2021	-1.54(-1.78, -1.3)	30.87	<0.001
		**Full Range**	**1990-2021**	**-2.86(-2.89, -2.83)**	**-48.1**	**<0.001**
	Female	0	1990-2000	-1.52(-1.55, -1.49)	-8.24	<0.001
		1	2000-2010	-2.72(-2.75, -2.68)	10.79	<0.001
		2	2010-2018	-1.87(-1.93, -1.82)	6.93	<0.001
		3	2018-2021	-0.97(-1.17, -0.77)	-36.03	<0.001
		**Full Range**	**1990-2021**	**-1.94(-1.97, -1.92)**	**-39.15**	**<0.001**
	Male	0	1990-2001	-3.79(-3.87, -3.71)	-5.49	<0.001
		1	2001-2010	-6.85(-6.97, -6.72)	7.45	<0.001
		2	2010-2018	-4.62(-4.79, -4.46)	-29.77	<0.001
		3	2018-2021	-2.96(-3.6, -2.31)	-17.67	<0.001
		**Full Range**	**1990-2021**	**-4.82(-4.9, -4.74)**	**-38.92**	**<0.001**
Japan	Both	0	1990-1994	-3.13(-3.32, -2.94)	-24.92	<0.001
		1	1994-1999	-2.23(-2.42, -2.04)	-61.19	<0.001
		2	1999-2004	-1.02(-1.21, -0.82)	-26.31	<0.001
		3	2004-2021	0.32(0.3, 0.34)	-3.38	0.003
		**Full Range**	**1990-2021**	**-0.76(-0.81, -0.71)**	**-8.61**	**<0.001**
	Female	0	1990-1998	-2.59(-2.7, -2.48)	-45.84	<0.001
		1	1998-2004	-0.89(-1.11, -0.67)	-13.59	<0.001
		2	2004-2012	0.7(0.56, 0.83)	-0.43	0.672
		3	2012-2021	0.31(0.21, 0.4)	-35.74	<0.001
		**Full Range**	**1990-2021**	**-0.58(-0.64, -0.52)**	**-115.25**	**<0.001**
	Male	0	1990-1995	-3.53(-3.73, -3.33)	-32.16	<0.001

		1	1995-2004	-1.94(-2.04, -1.84)	-5.82	<0.001
		2	2004-2013	-0.27(-0.38, -0.17)	-53.03	<0.001
		3	2013-2021	0.37(0.27, 0.48)	-24.44	<0.001
		**Full Range**	**1990-2021**	**-1.13(-1.18, -1.07)**	**-83.85**	**<0.001**
South Korea	Both	0	1990-1994	-5.03(-5.43, -4.62)	-53.07	<0.001
	Both	1	1994-2006	-2.61(-2.69, -2.52)	-12.78	<0.001
		2	2006-2016	-1.46(-1.58, -1.35)	-41.29	<0.001
		3	2016-2021	-0.45(-0.72, -0.17)	-3.9	0.001
		**Full Range**	**1990-2021**	**-2.21(-2.29, -2.13)**	**-35.44**	**<0.001**
	Female	0	1990-1993	-3.21(-3.97, -2.45)	-13.46	<0.001
		1	1993-2008	-1.53(-1.6, -1.47)	-21.65	<0.001
		2	2008-2018	-0.86(-0.99, -0.73)	-0.93	0.365
		3	2018-2021	-0.14(-0.84, 0.55)	-33.78	<0.001
		**Full Range**	**1990-2021**	**-1.35(-1.45, -1.24)**	**-8.13**	**<0.001**
	Male	0	1990-1994	-8.4(-8.87, -7.94)	-85.62	<0.001
		1	1994-2006	-5.73(-5.83, -5.63)	-11.6	<0.001
		2	2006-2015	-2.71(-2.88, -2.53)	-38.05	<0.001
		3	2015-2021	-0.79(-1.08, -0.51)	-27.07	<0.001
		**Full Range**	**1990-2021**	**-4.27(-4.37, -4.18)**	**-48.17**	**<0.001**

* APC (annual percentage change) for each period and AAPC (average annual percentage change) for the full range.

Abbreviations: YLDs, Years Lived with Disability.

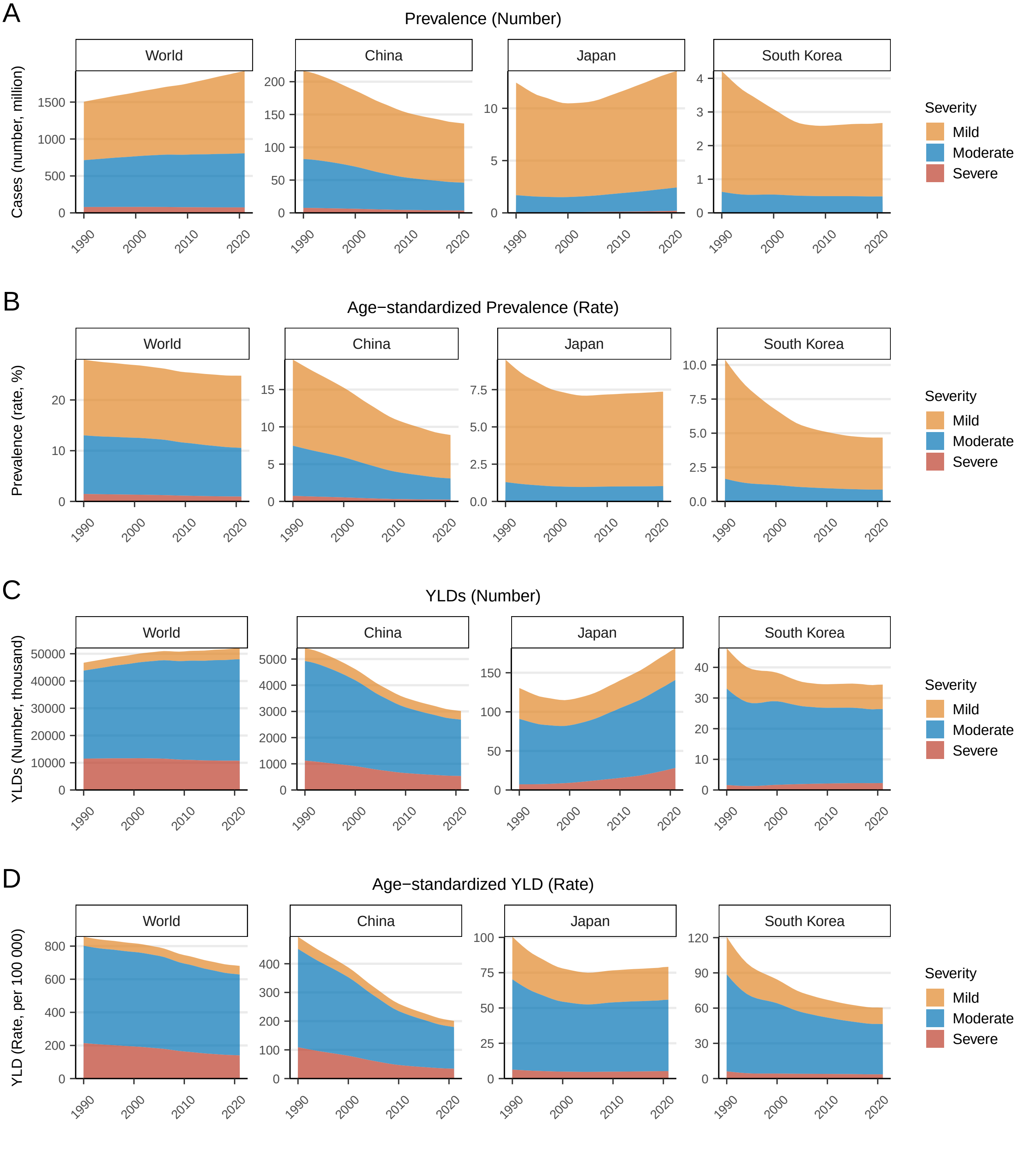
A
Prevalence (Number)
Cases (number, million)
B
Age−standardized Prevalence (Rate)
Prevalence (rate, %)
C
YLDs (Number)
YLDs (Number, thousand)
D
Age−standardized YLD (Rate)
YLD (Rate, per 100 000)
World
China
Japan
South Korea
Severity
Mild
Moderate
Severe

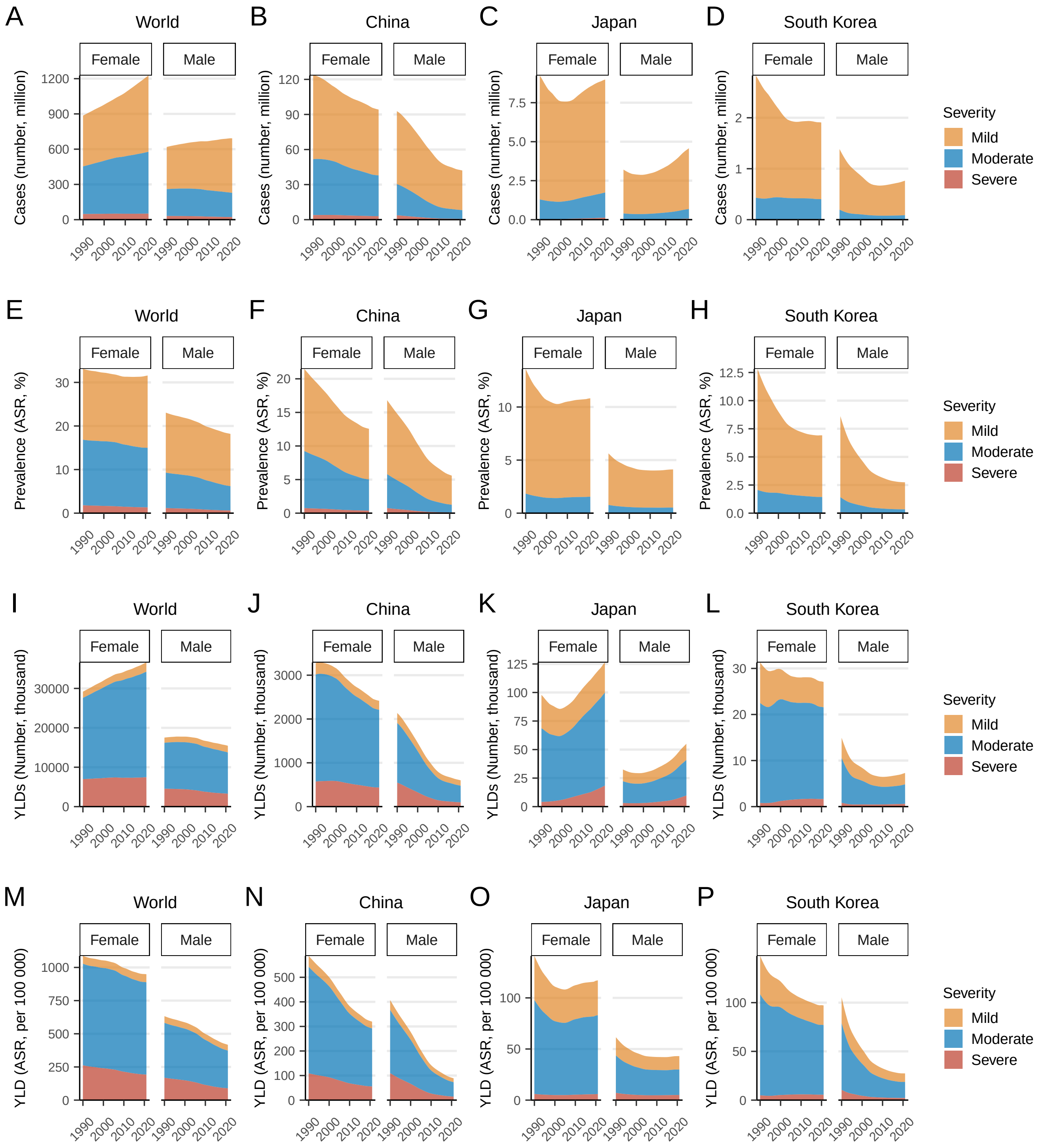

A
World
B
China
C
Japan
D
South Korea
Female
Male
Cases (number, million)
E
F
G
H
Prevalence (ASR, %)
I
J
K
L
YLDs (Number, thousand)
M
N
O
P
YLD (ASR, per 100 000)
1990
2000
2010
2020
Severity
Mild
Moderate
Severe

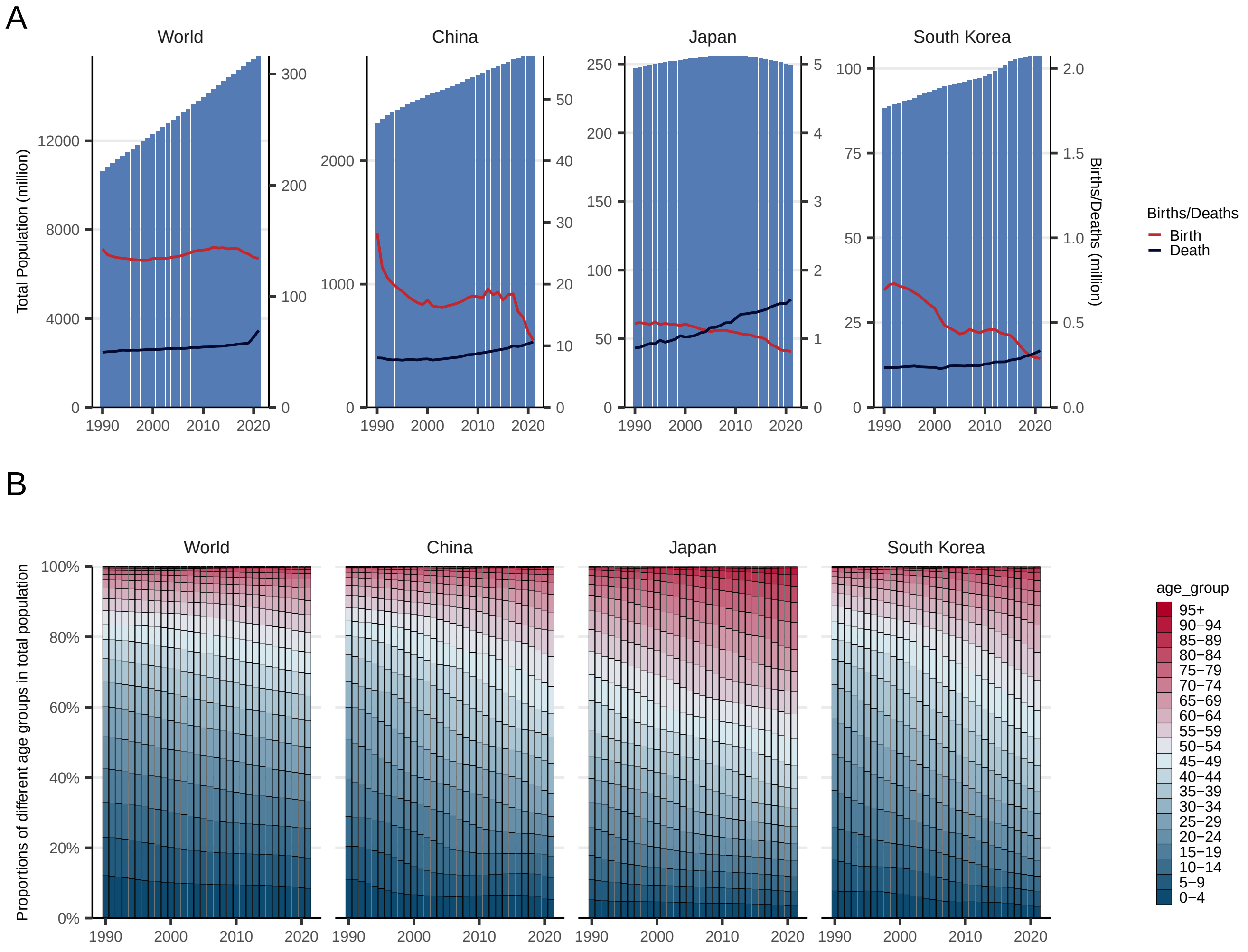

A
World
China
Japan
South Korea
Total Population (million)
Births/Deaths (million)
Births/Deaths
Birth
Death
B
World
China
Japan
South Korea
Proportions of different age groups in total population
age_group
95+
90–94
85–89
80–84
75–79
70–74
65–69
60–64
55–59
50–54
45–49
40–44
35–39
30–34
25–29
20–24
15–19
10–14
5–9
0–4
